# Epidermal growth factor and transforming growth factor alpha concentrations in BPH and cancer of the prostate: their relationships with tissue androgen levels.

**DOI:** 10.1038/bjc.1993.26

**Published:** 1993-01

**Authors:** Y. Yang, G. D. Chisholm, F. K. Habib

**Affiliations:** University Department of Surgery (WGH), Western General Hospital, Edinburgh, Scotland, UK.

## Abstract

We measured immunoreactive EGF and TGF alpha in prostate tissue extracts obtained from 19 patients with benign prostatic hyperplasia (BPH) and 19 with cancer of the prostate (CaP). Whilst both BPH and CaP expressed EGF (BPH = 195.61 +/- 19.94 ng g-1 protein; CaP = 235.60 +/- 24.45 ng g-1 protein) and TGF alpha (BPH = 92.57 +/- 7.60 ng g-1 protein; CaP = 100.73 +/- 15.47 ng g-1 protein) in equal concentrations, the levels of EGF in any tissue extract were on average twice those of TGF alpha. Furthermore analysis of the individual growth factor data revealed a direct correlation between EGF and TGF alpha in both BPH (r = 0.72, P < 0.001) and CaP (r = 0.69, P < 0.001). When the tumours were classified according to their Gleason score, a slight but significant increase in growth factor concentrations was noted as the tumour became less differentiated. We also measured the concentrations of testosterone and dihydrotestosterone (DHT) in prostate extracts with a view of elucidating the relationship between androgen and growth factors in this gland. There was a small positive correlation only between testosterone and EGF (r = 0.62, P < 0.05) and testosterone and TGF alpha (r = 0.61, P < 0.05) in CaP. The absence of any similar correlation in BPH where DHT becomes the predominant hormone may suggest an indirect role for testosterone in the regulation of growth factor production.


*Br. J. Cancer* (1993), **67**, 152–155 © Macmillan Press Ltd., 1993

### Epidermal growth factor and transforming growth factor α concentrations in BPH and cancer of the prostate: their relationships with tissue androgen levels

Y. Yang, G.D. Chisholm & F.K. Habib

*University Department of Surgery (WGH), Western General Hospital, Crewe Road, Edinburgh EH4 2XU, Scotland, UK.*

**Summary** We measured immunoreactive EGF and TGFα in prostate tissue extracts obtained from 19 patients with benign prostatic hyperplasia (BPH) and 19 with cancer of the prostate (CaP). Whilst both BPH and CaP expressed EGF (BPH = 195.61 ± 19.94 ng $g^{-1}$ protein; CaP = 235.60 ± 24.45 ng $g^{-1}$ protein) and TGFα (BPH = 92.57 ± 7.60 ng $g^{-1}$ protein; CaP = 100.73 ± 15.47 ng $g^{-1}$ protein) in equal concentrations, the levels of EGF in any tissue extract were on average twice those of TGFα. Furthermore analysis of the individual growth factor data revealed a direct correlation between EGF and TGFα in both BPH ($r = 0.72$, $P < 0.001$) and CaP ($r = 0.69$, $P < 0.001$). When the tumours were classified according to their Gleason score, a slight but significant increase in growth factor concentrations was noted as the tumour became less differentiated. We also measured the concentrations of testosterone and dihydrotestosterone (DHT) in prostate extracts with a view of elucidating the relationship between androgen and growth factors in this gland. There was a small positive correlation only between testosterone and EGF ($r = 0.62$, $P < 0.05$) and testosterone and TGFα ($r = 0.61$, $P < 0.05$) in CaP. The absence of any similar correlation in BPH where DHT becomes the predominant hormone may suggest an indirect role for testosterone in the regulation of growth factor production.

Since the realisation that androgens are not alone in influencing the growth and regulation of the prostate, several investigations into the role of growth factors and their relationship with androgens have been undertaken. Initially, studies on the normal rat prostate demonstrated that the receptors for epidermal growth factor (EGF) were down regulated by androgens (Traish & Wotiz, 1987; St. Arnaud *et al.*, 1988). Subsequently experiments on androgen responsive prostate cancer cell lines revealed relatively low numbers of EGF receptors whilst androgen unresponsive cells expressed higher EGF receptor numbers (MacDonald & Habib, 1992). The correlation between EGF receptor expression and endocrine status suggest that steroid hormones might influence the response to growth factors, by altering growth factor receptor expression, but this response may also reflect the species from which the prostate cells were derived. Receptors for EGF have also been identified in human benign hyperplastic prostate (BPH) but their concentrations were markedly depleted in the poorly differentiated prostate cancer (Maddy *et al.*, 1989). Significantly, this loss in EGF-receptor expression is manifested at a time when the prostate tumour exhibits a reduced sensitivity to steroid hormones. It is not clear whether these two events are interrelated but the progression to an androgen independent state may, in part, be due to a reduced need for exogenous mitogens because of the the autologous production of growth factors (Sporn & Roberts, 1985). Transforming growth factor alpha (TGFα) is a polypeptide growth factor which has been reported to modulate autocrine growth in several tumour systems (Bates *et al.*, 1986; Hofer *et al.*, 1991; Lloyd *et al.*, 1992; MacDonald *et al.*, 1990) but no one has so far related the presence of this growth factor in BPH and cancer of the prostate to the tissular concentrations of EGF and prostate androgens. The purpose of this study is to investigate some of these relationships.

Correspondence: F.K. Habib, University Department of Surgery (WGH), Western General Hospital, Crewe Road, Edinburgh EH4 2XU, Scotland, UK.
Received 18 February 1992; and in revised form 12 August 1992.

### Materials and methods

### *Prostate tissue*

Prostate tissue was obtained at the time of transurethral resection from 19 patients with BPH and 19 patients with prostatic cancer. The tissue was transported immediately to the laboratory chopped into 1–3 g pieces washed in 2 × 150 mM sodium chloride solution and dry blotted. Several sections from each specimen were sent for histological examination and grading of the cancerous tissue by the Gleason system (Gleason, 1977). The remainder of the tissue was snap frozen in liquid nitrogen and finally pulverised in a micro-dismembrator (Braun AG, Melsungen, Germany). The powdered tissue was then lyophilised to dryness and stored at − 70°C until needed. For growth factor studies 70 mg of the lyophilised material was used whereas only 50 mg were required for steroid measurements.

### *Extraction of growth factors*

Lyophilised samples were resuspended in 1 ml buffer (50 mmol $l^{-1}$ Tris, 5 mol $l^{-1}$ of phenyl-methylsulphonyl fluoride, 2 mmol $l^{-1}$ of EDTA pH 7.4) containing 1,000 counts of either $^{125}$I-EGF or $^{125}$I-TGFα to monitor manipulative losses. The samples were homogenised with a glass/glass Dounce Homogeniser and left on ice for 1 h to equilibrate. The homogenates were then centrifuged, the supernatants saved and the resultant pellets were resuspended in 2 ml of enzyme mixture (230 U $ml^{-1}$ of collagenase, 125 U $ml^{-1}$ of hyaluronidase in 150 mmol $l^{-1}$ of NaCl) and incubated overnight at 37°C; these conditions were found to be optimal and ensured maximum growth factor recovery. The digested pellets were subsequently sonicated (six cycles of 20 s with 1 min cooling interval at an amplitude of 22 μ; MSE soniprep 150) the resultant mixture was centrifuged as before, the supernatant saved and the pellet was resuspended in 2 ml Tris buffer. Homogenisation, centrifugation and resuspension were then repeated twice with the supernatants always being saved. The supernatants were then pooled and the recoveries were assessed. The mean recovery of $^{125}$I-EGF added at the beginning of the extraction procedure was 72 ± 6% whilst that of $^{125}$I-TGFα was 69 ± 8%. The supernatants were finally snap frozen, lyophilised to dryness and stored at − 70° until required.

*Radio-immunoassays for hEGF and hTGFα*

The concentrations of immunoreactive hEGF and hTGFα in the lyophilised samples were determined by a liquid phase competitive RIA. Briefly, the lyophilised material was reconstituted in 700 μl of PBS (0.02 mol $l^{-1}$ of $Na_2HPO4.2H_2O$, 0.02 mol $l^{-1}$ $Na_2HPO4.2H_2O$, 0.9% NaCl, 3.7 g $l^{-1}$ of EDTA, 1 g $l^{-1}$ of BSA, pH 7.4) and the assays were performed in triplicate employing 100 μl of the reconstituted lyophilised material. The radio immunoassay for hEGF was as described previously (MacDonald *et al.*, 1990). No cross reaction was found with transforming growth factor-α, which exerts all of its biological effects via the EGF receptor, up to concentrations of 1 μg $ml^{-1}$. Cross reactivity of this antiserum with a large variety of other substances has been tested by other investigators (Gregory *et al.*, 1979; Jaspar *et al.*, 1985). TGFα content was analysed using a commercial kit (Peninsula Laboratory Europe Ltd, St Helens, Merseyside) with rTGF1 as radioiodinated tracer and hTGFα as reference standard. Antisera to the rTGF1 was raised in rabbits and purchased from Peninsula Laboratory Europe Ltd. The rabbit anti-rat TGF1 antisera recognises both mouse and hTGFα but does not crossreact with either mouse or hEGF. Half maximal inhibition of binding of the $^{125}$I-peptide to the antibody occurred at 140 pg/tube.

*Measurement of androgens*

Specific radioimmunoassays for testosterone and dihydrotestosterone (DHT) were carried out after a single separation on Gelman ITLC plates using an antiserum against dihydrotestosterone (Guildhay Antisera Ltd, Guildford, Surrey). The extraction of the androgens from the tissue, chromatographic separation, radioimmunoassay and reliability of the method have already been described in detail (Habib *et al.*, 1976; Houston *et al.*, 1985). Briefly 50 mg of the lyophilised powdered tissue extract was reconstituted in 2 ml of Tris HCl buffer containing 1,000 cpm radioactive steroids for each of the androgens to be assayed for recovery. Following equilibration, the steroids were extracted three times with diethylether and the pooled extracts were dried down, reconstituted in 50 μl ethanol and separated on thin layer chromatography plates. The steroids were subsequently extracted, reconstituted in PBS buffer, recovery was assessed and the radioimmunoassay carried out.

*Protein determination*

Protein was estimated by the method of Bradford (1976) using bovine serum albumin as standard.

*Data analysis*

All analysis were performed in triplicate and the data has been presented as mean ± standard error of the mean. Differences in growth factors and androgen concentrations were tested for statistical significance by student *t*-test. Correlation of the degree of association for any two parameters was determined by calculation of the correlation co-efficient ($r$): $r$ values presenting a probability of $<0.05$ were considered to be statistically significant.

### Results

Figure 1 demonstrates the relative concentrations of EGF and TGFα in tissue extracts obtained from patients with BPH ($n = 19$) and prostate cancer ($n = 19$). The mean ± s.e.m. values (ng $g^{-1}$ protein) for EGF (195.61 ± 19.94) and TGFα (92.57 ± 7.60) in the BPH specimens were slightly but not significantly ($P > 0.3$) lower than those measured in the cancer tissue extracts (EGF = 235.60 ± 24.45; TGFα = 100.73 ± 15.47).

Additionally the EGF/TGFα ratios in both tissue types were found to be of the same order of magnitude (mean

**Figure 1** Comparison of immunoreactive EGF and TGFα in tissue extracts obtained from 19 patients with BPH and 19 patients with cancer of the prostate (CaP). The bars represent the mean values of all extracts within each group ± s.e.m.

value for BPH = 2.12; CaP = 2.34) with the EGF concentration in each tissue extract being approximately twice the TGFα concentration.

Analysis of the individual EGF and TGFα values in BPH and CaP by-linear regression (Figures 2 and 3) resulted in a direct correlation between EGF and TGFα in both BPH ($r = 0.72$, $P < 0.001$) and CaP ($r = 0.69$, $P < 0.001$).

Furthermore when 13 of the tumours were classified according to their histological grade (Gleason score: primary- + secondary pattern) and these were in turn correlated to their growth factor concentrations, a significant correlation was found between the Gleason score and EGF ($r = 0.65$, $P < 0.02$) and the Gleason score and TGFα ($r = 0.57$, $P < 0.05$) with the concentration of the growth factors in the tissue extracts increasing as the tumour becomes less differentiated (Table I).

Testosterone and DHT concentrations were also measured in the tissue extracts. These measurements confirmed our earlier findings revealing significantly higher levels of testosterone in CaP (289.26 ± 76.9, $n = 12$) when compared to BPH (94.02 ± 14.4, $n = 19$) whilst DHT concentrations were significantly higher in BPH (201.64 ± 38.4) than in CaP tissue extracts (79.11 ± 27.8). The differences between the two groups were statistically significant ($P < 0.01$).

Attempts to correlate the steroid hormone levels with the growth factor concentrations employing linear regression

**Table I** Effect of tumour differentiation on tissue EGF and TGFα concentrations

| *Gleason score* | *EGF (ng g⁻¹ protein)* | *TGFα (ng g⁻¹ protein)* |
|---|---|---|
| 2–4 | 135.08[a] (1) | 32.24 (1) |
| 5–7 | 203.78±24.08[b] (8) | 91.62±12.95 (8) |
| 8–10 | 359.18±54.06 (4) | 123.38±41.6 (4) |

[a]Parentheses denote number of tumours in each group. [b]Mean ± s.e.m.

**Figure 2** Relationship between the concentration of immunoreactive EGF and that of immunoreactive TGFα in prostate tissue extracts from 19 patients with benign prostatic hyperplasia ($r = 0.72$, $P < 0.001$).

**Figure** 3 Relationship between the concentrations of immunoreactive EGF and TGFα in the tissue extracts obtained from 19 patients with carcinoma of the prostate ($r = 0.69$, $P<0.001$).

yielded positive correlation only between testosterone and EGF ($r = 0.62$, $P<0.05$) and testosterone and TGFα ($r = 0.61$, $P<0.05$) in CaP (Figure 4). No similar correlation was found in BPH or indeed between DHT and the growth factors for both tissue extracts.

### Discussion

The present study confirms the presence of immunoreactive EGF and TGFα in tissue extracts from hyperplastic and neoplastic human prostates. These findings are consistent with the reports on the production of EGF-related peptides in human prostate tissue (Gregory *et al.*, 1987; Fowler *et al.*, 1986) and TGFα-like molecules by prostate cancer cell lines (Connolly & Rose, 1989; MacDonald *et al.*, 1990; Hofer *et al.*, 1991). TGFα was originally characterised by its ability to induce phenotypic transformation of normal cells (Sporn & Roberts, 1985) and the elevated expression of this growth factor in transformed cells has been reported (Marquardt *et al.*, 1983). However, TGFα has since been found also in a variety of other normal as well as neoplastic tissues (Coffey *et al.*, 1987; Gomella *et al.*, 1989; Lloyd *et al.*, 1992), suggesting a possible role for this growth factor in normal cell physiology.

The expression of TGFα in human benign prostate tissue has not previously been reported. In the current study, the TGFα concentrations in BPH were compatible with those measured in other tissues (Liu *et al.*, 1990). It was also of interest to note that there was no difference in the TGFα levels between BPH and prostate cancers even though there was a marginal increase in TGFα expression as the tumour became less differentiated. Significantly however, this increase in TGFα concentrations was manifested at a time when the expression of the EGF-receptor is down regulated (Maddy *et al.*, 1989) thus raising further the possibility that TGFα may be excerting its biological effects by interacting with receptor sites other than those associated with EGF (Winkler *et al.*, 1989). Although one should not exclude the possibility that higher levels of EGF-R activation can also result in more rapid receptor turnover and/or down regulation of receptor expression.

Until recently TGFα was thought to be the counterpart of EGF in cancer, produced by the neoplastic prostate cell at the expense of EGF as part of an autocrine regulatory mechanism to reduce the need of these cells for exogenous mitogens (MacDonald *et al.*, 1990; Hofer et al., 1991). However the presence of immunoreactive EGF in extracts from BPH and cancer at equivalent concentrations highlights, once more, the complexity of the biological actions of these two growth factors in the human prostate. Additionally, the close association found between TGFα and EGF in the two tissue types suggests that the mechanism responsible for the secretion of one of these growth factors may also be responsible for controlling the production of the other. Whether both growth factors act in synergy to induce neoplastic transformation in the human prostate still remains to be clarified. In view of the increasing interest in the role of growth factors in endocrine related tumours (Bates *et al.*, 1986; Lai *et al.*, 1989; MacDonald & Habib, 1992; Perheentupa *et al.*, 1984) attempts were also made to establish the relationship between steroid hormone concentrations and the growth factors in the prostate extracts. Whilst there was some correlation between testosterone and TGFα and EGF in prostate cancer, the absence of a parallel association in BPH where DHT is the active steroid may possibly suggest an indirect role for testosterone in the regulation of prostatic EGF and TGFα. Though this relationship will need to be confirmed in a more dynamic system, the production of growth factors in some tissues has been shown to be sensitive to sex hormones. This has been witnessed in a number of systems including the submaxillary gland where testosterone increases EGF levels (Perheentupa *et al.*, 1984), in various human cancer cell lines where oestradiol influences the production of EGF related polypeptides (Lippman *et al.*, 1986) and in breast cyst fluid where androgens modulate EGF concentrations (Lai *et al.*, 1989).

**Figure** 4 The correlation between the concentrations of testosterone and EGF **a**, and testosterone and TGFα **b**, as measured in tissue extracts obtained from 12 patients with cancer of the prostate. Regression lines are shown with correlation coefficients ($r$).

### References

BATES, S., MACMANAWAY,M., LIPPMAN, M. & DICKSON, R. (1986). Characterisation of oestrogen-responsive transforming activity in human breast cancer cell lines. *Cancer Res.*, **46**, 1707-1713.

BRADFORD, M.M. (1976). A rapid and sensitive method for the quantification of microgram quantities of protein utilizing the principle of protein dye binding. *Anal. Biochem.*, **72**, 248-254.

COFFEY, R.J., DERYNCK, R., WILCOX, J.N., BRINGMANN, T.S., GOUSTIN, A.S., MOSES, H.L. & PITTLEKOW, M.R. (1987). Production and auto-induction of transforming growth factor-alpha in human keratinocytes. *Nature*, **328**, 817-820.

CONNOLLY, J.M. & ROSE, D.P. (1989). Secretion of epidermal growth factor related polypeptides by the DU145 human prostate cancer cell line. *Prostate*, **15**, 177-186.

FOWLER, J.E., LAU, J.L.T., GHOSH, L., MILLS, S.E. & MOUNZER, A. (1988). Epidermal growth factor and prostatic carcinoma: an immunohistochemical study. *J. Urol.*, **139**, 857-861.

GLEASON, D.F. (1977). Histologic grading and clinical staging of prostatic cancer. In *Urologic Pathology: the Prostate*. Tannenbaum, M. (ed.) pp. 171-198. Lea & Febiger: New York.

GOMELLA, L.G., SARGENT, E.R., WADE, T.P., ANGLARD, P., MARSTON, LINEHAN, W. & KUSID, A. (1989). Expression of transforming growth factor alpha in normal human adult kidney and enhanced expression of transforming growth factors alpha and beta 1 in renal cell carcinoma. *Cancer Res.*, **49**, 6972-6975.

GREGORY, H., HOLMES, J.E. & WILLSHIRE, I.R. (1979). Urogastrone/epidermal growth factor. In Jaffe, B.M. & Behrman, H.R. (eds), *Methods of Hormone Radioimmunoassay*, 2nd edit, pp. 927-937. Academic Press: New York.

GREGORY, H., WILLSHIRE, I.R., KAVANAGH, J.P., BLACKLOCK, N.J., CHOWDURY, S. & RICHARDS, R.C. (1986). Urogastrone-epidermal growth factor concentrations in prostatic fluid of normal individuals and patients with benign prostatic hypertrophy. *Clin. Sci.*, **70**, 359-363.

HABIB, F.K., LEE, I.R., STITCH, S.R. & SMITH, P.H. (1976). Androgen levels in the plasma and prostatic tissues of patients with benign hypertrophy and carcinoma of the prostate. *J. Endocrinol.*, **71**, 99-107.

HOFER, D.R., SHERWOOD, E.R., BROMBERG, W.D., MENDELSOHN, J., LEE, C. & KOZLOWSKI, J.M. (1991). Autonomous growth of androgen-independent human prostatic carcinoma cells: Role of transforming growth factor alpha. *Cancer Res.*, **51**, 2780-2785.

HOUSTON, B., CHISHOLM, G.D. & HABIB, F.K. (1985). Solubilization of human prostatic 5α-reductase. *J. Steroid Biochem.*, **22**, 461-467.

JASPAR, S.M. & FRANCHIMRET, P. (1985). Radioimmunoassay of human epidermal growth factor in human breast cyst fluid. *Eur. J. Cancer Clin. Oncol.*, **21**, 1343-1348.

LAI, L.C., GHILCHIK, M.W., SHAIKH, N.A., REED, M.J. & JAMES, V.H.T. (1989). Relationship between epidermal growth factor and dehydroepiandrosterone and its sulphate in breast cyst fluid. *Br. J. Cancer*, **60**, 320-323.

LLOYD, S.N., BROWN, I.L. & LEAKE, R.E. (1992). Transforming growth factor α expression in benign and malignant human prostatic disease. *Int. J. Biol. Markers*, **7**, 27-34.

LIPPMAN, M.E., DICKSON, R.B., KASID, A., GELMANN, E., DAVIDSON, N., MCMANAWAY, M., HUFF, K., BRONZERT, D., BATES, S., SWAIN, S. & KNABBE, C. (1986). Autocrine and paracrine growth regulation of human breast cancer. *J. Steroid Biochem.*, **24**, 147-154.

LIU, C., WOO, A. & TSAO, M.S. (1990). Expression of transforming growth factor-alpha in primary human colon and lung carcinomas. *Br. J. Cancer*, **62**, 425-429.

MACDONALD, A., CHISHOLM, G.D. & HABIB, F.K. (1990). Production and response of a human prostatic cancer line to transforming growth factor-like molecules. *Br. J. Cancer*, **62**, 579-584.

MACDONALD, A. & HABIB, F.K. (1992). Divergent responses to epidermal growth factor in hormone sensitive and insensitive human prostate cancer cell lines. *Br. J. Cancer*, **65**, 177-182.

MADDY, S.Q., CHISHOLM, G.D., BUSUTTIL, A. & HABIB, F.K. (1989). Epidermal growth factor receptors in human prostate cancer: Correlation with histological differentiation of the tumour. *Br. J. Cancer*, **60**, 41-44.

MARQUARDT, H., HUNKAPILLER, M.W., HOOD, L.E., TWARDZIK, D.R., DELARCA, J.E., STEPHENSON, T.R. & TODARO, G.T. (1983). Transforming growth factors produced by retrovirus-transformed rodent fibroblasts and human melanoma cells: Amino acid sequence homology with epidermal growth factor. *Proc. Natl Acad. Sci. USA*, **80**, 4684-4688.

PERHEENTUPA, J., LAKSHMANAN, J., HOATH, S.B. & FISHER, D.A. (1984). Hormonal modulation of mouse plasma concentration of epidermal growth factor. *Acta Endocrinol.*, **107**, 571-576.

ST-ARNAUD, R., POYET, P., WALKER, P. & LABRIE, F. (1988). Androgens modulate epidermal growth factor receptor levels in the rat ventral prostate. *Mol. Cell Endocrinol.*, **56**, 21-27.

SPORN, M.B. & ROBERTS, A.B. (1985). Autocrine growth factors and cancer. *Nature*, **313**, 745-747.

TRAISH, A.M. & WOTIZ, H.H. (1987). Prostatic epidermal growth factor receptors and their regulation by androgens. *Endocrinol.* **121**. 1461-1467.

WINKLER, M.E., O'CONNOR, L., WINGATE, M. & FENDLY, B. (1989). Epidermal growth factor and transforming growth factor alpha bind differently to the epidermal growth factor receptor. *Biochem.*, **28**, 6373-6378.


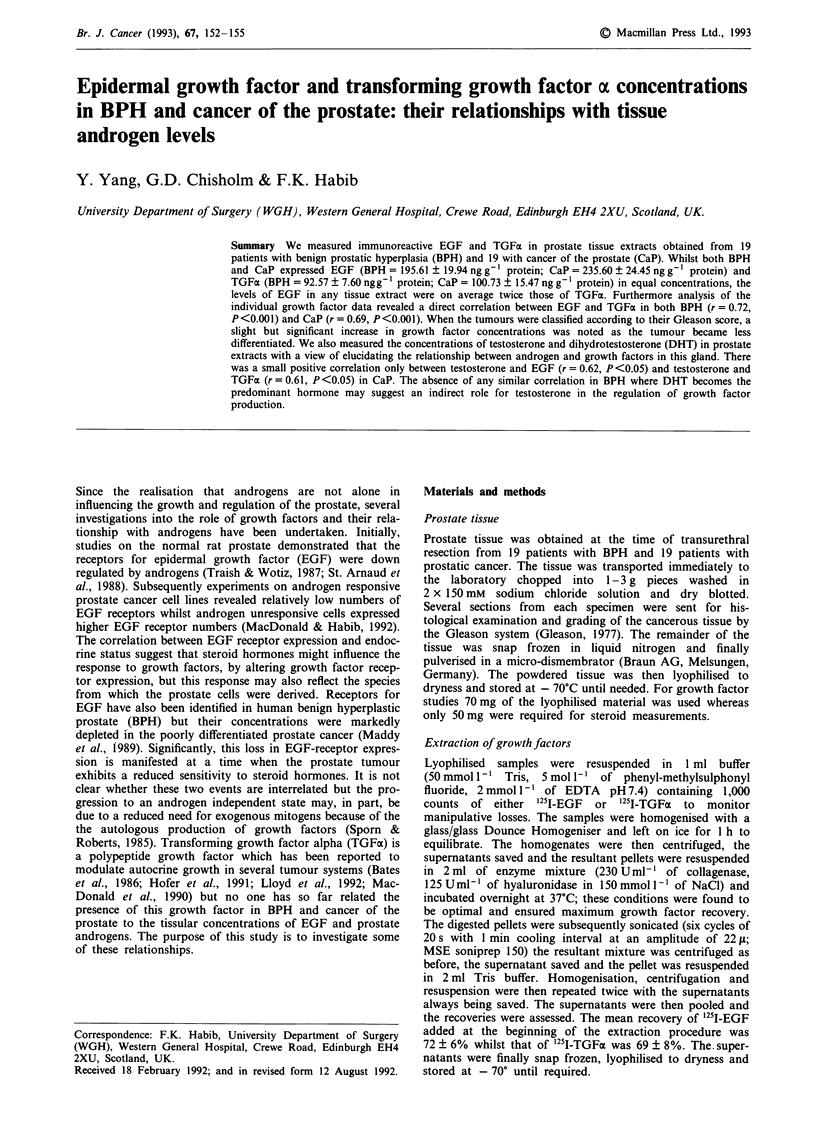

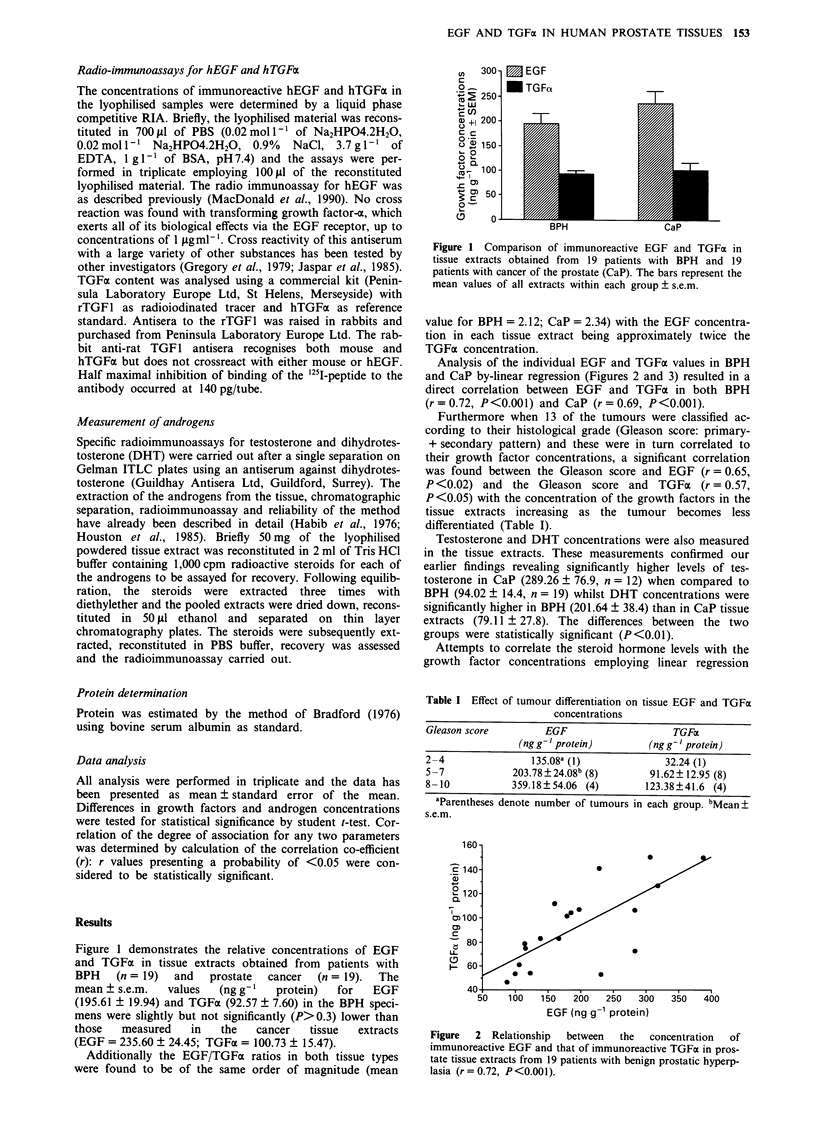

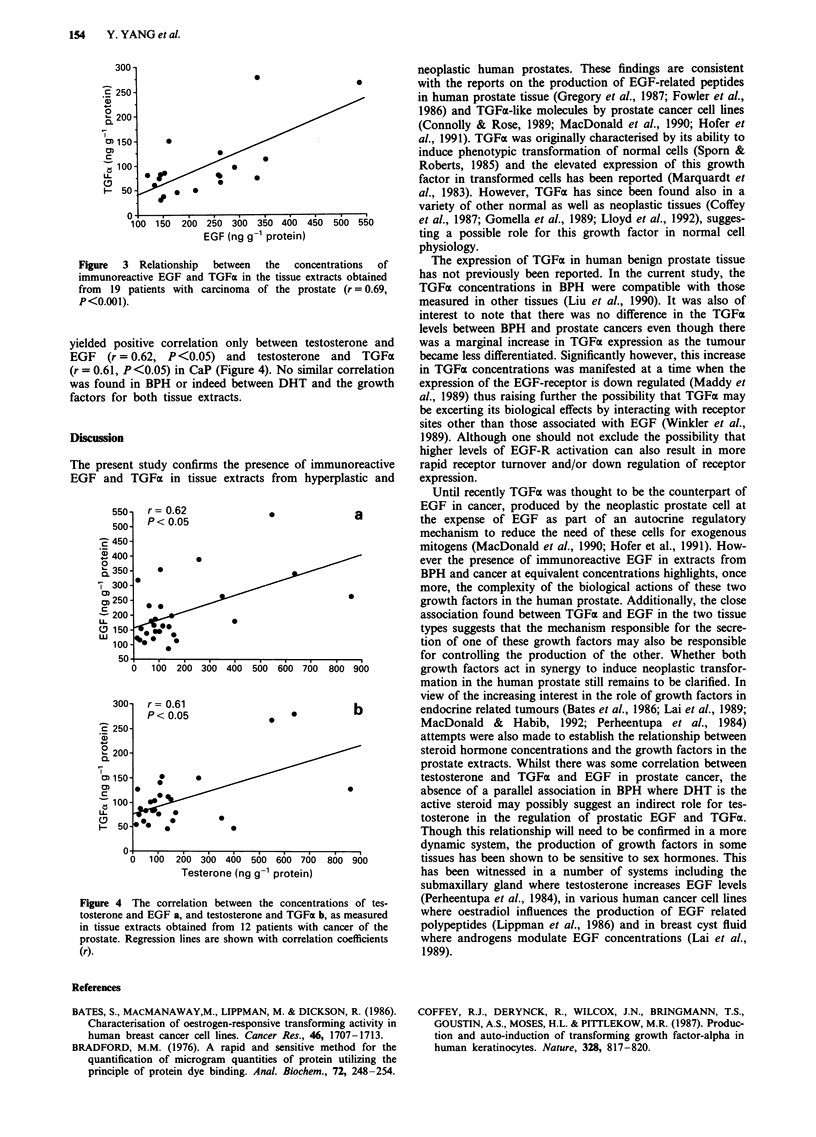

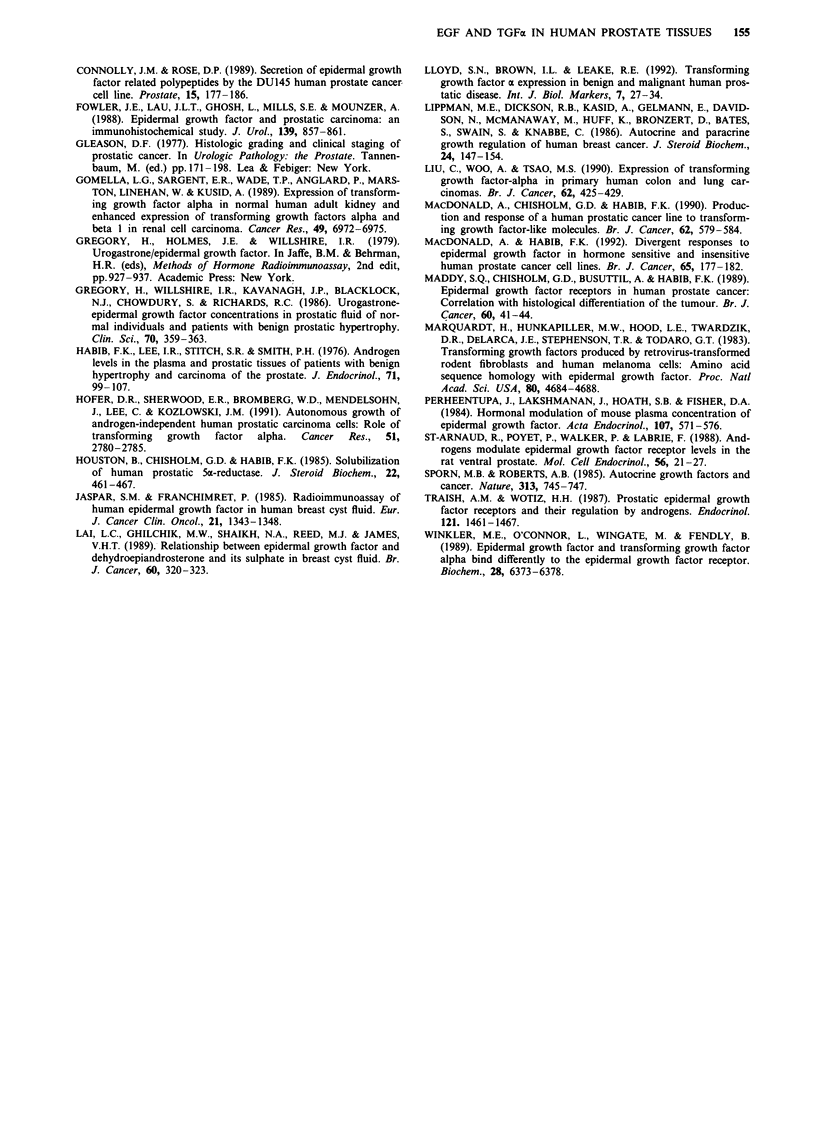


## References

[OCR_00435] Bradford M. M. (1976). A rapid and sensitive method for the quantitation of microgram quantities of protein utilizing the principle of protein-dye binding.. Anal Biochem.

[OCR_00440] Coffey R. J., Derynck R., Wilcox J. N., Bringman T. S., Goustin A. S., Moses H. L., Pittelkow M. R. Production and auto-induction of transforming growth factor-alpha in human keratinocytes.. Nature.

[OCR_00448] Connolly J. M., Rose D. P. (1989). Secretion of epidermal growth factor and related polypeptides by the DU 145 human prostate cancer cell line.. Prostate.

[OCR_00430] Dickson R. B., Bates S. E., McManaway M. E., Lippman M. E. (1986). Characterization of estrogen responsive transforming activity in human breast cancer cell lines.. Cancer Res.

[OCR_00453] Fowler J. E., Lau J. L., Ghosh L., Mills S. E., Mounzer A. (1988). Epidermal growth factor and prostatic carcinoma: an immunohistochemical study.. J Urol.

[OCR_00463] Gomella L. G., Sargent E. R., Wade T. P., Anglard P., Linehan W. M., Kasid A. (1989). Expression of transforming growth factor alpha in normal human adult kidney and enhanced expression of transforming growth factors alpha and beta 1 in renal cell carcinoma.. Cancer Res.

[OCR_00476] Gregory H., Willshire I. R., Kavanagh J. P., Blacklock N. J., Chowdury S., Richards R. C. (1986). Urogastrone-epidermal growth factor concentrations in prostatic fluid of normal individuals and patients with benign prostatic hypertrophy.. Clin Sci (Lond).

[OCR_00483] Habib F. K., Lee I. R., Stitch S. R., Smith P. H. (1976). Androgen levels in the plasma and prostatic tissues of patients with benign hypertrophy and carcinoma of the prostate.. J Endocrinol.

[OCR_00489] Hofer D. R., Sherwood E. R., Bromberg W. D., Mendelsohn J., Lee C., Kozlowski J. M. (1991). Autonomous growth of androgen-independent human prostatic carcinoma cells: role of transforming growth factor alpha.. Cancer Res.

[OCR_00496] Houston B., Chisholm G. D., Habib F. K. (1985). Solubilization of human prostatic 5 alpha-reductase.. J Steroid Biochem.

[OCR_00501] Jaspar J. M., Franchimont P. (1985). Radioimmunoassay of human epidermal growth factor in human breast cyst fluid.. Eur J Cancer Clin Oncol.

[OCR_00506] Lai L. C., Ghilchik M. W., Shaikh N. A., Reed M. J., James V. H. (1989). Relationship between epidermal growth factor and dehydroepiandrosterone and its sulphate in breast cyst fluid.. Br J Cancer.

[OCR_00519] Lippman M. E., Dickson R. B., Kasid A., Gelmann E., Davidson N., McManaway M., Huff K., Bronzert D., Bates S., Swain S. (1986). Autocrine and paracrine growth regulation of human breast cancer.. J Steroid Biochem.

[OCR_00524] Liu C., Woo A., Tsao M. S. (1990). Expression of transforming growth factor-alpha in primary human colon and lung carcinomas.. Br J Cancer.

[OCR_00512] Lloyd S. N., Brown I. L., Leake R. E. (1992). Transforming growth factor-alpha expression in benign and malignant human prostatic disease.. Int J Biol Markers.

[OCR_00529] MacDonald A., Chisholm G. D., Habib F. K. (1990). Production and response of a human prostatic cancer line to transforming growth factor-like molecules.. Br J Cancer.

[OCR_00534] MacDonald A., Habib F. K. (1992). Divergent responses to epidermal growth factor in hormone sensitive and insensitive human prostate cancer cell lines.. Br J Cancer.

[OCR_00539] Maddy S. Q., Chisholm G. D., Busuttil A., Habib F. K. (1989). Epidermal growth factor receptors in human prostate cancer: correlation with histological differentiation of the tumour.. Br J Cancer.

[OCR_00545] Marquardt H., Hunkapiller M. W., Hood L. E., Twardzik D. R., De Larco J. E., Stephenson J. R., Todaro G. J. (1983). Transforming growth factors produced by retrovirus-transformed rodent fibroblasts and human melanoma cells: amino acid sequence homology with epidermal growth factor.. Proc Natl Acad Sci U S A.

[OCR_00553] Perheentupa J., Lakshmanan J., Hoath S. B., Fisher D. A. (1984). Hormonal modulation of mouse plasma concentration of epidermal growth factor.. Acta Endocrinol (Copenh).

[OCR_00563] Sporn M. B., Roberts A. B. Autocrine growth factors and cancer.. Nature.

[OCR_00558] St-Arnaud R., Poyet P., Walker P., Labrie F. (1988). Androgens modulate epidermal growth factor receptor levels in the rat ventral prostate.. Mol Cell Endocrinol.

[OCR_00567] Traish A. M., Wotiz H. H. (1987). Prostatic epidermal growth factor receptors and their regulation by androgens.. Endocrinology.

[OCR_00572] Winkler M. E., O'Connor L., Winget M., Fendly B. (1989). Epidermal growth factor and transforming growth factor alpha bind differently to the epidermal growth factor receptor.. Biochemistry.

